# An updated PREDICT breast cancer prognostic model including the benefits and harms of radiotherapy

**DOI:** 10.1038/s41523-024-00612-y

**Published:** 2024-01-15

**Authors:** Isabelle Grootes, Gordon C. Wishart, Paul David Peter Pharoah

**Affiliations:** 1 Department of Oncology, School of Clinical Medicine, University of Cambridge, Cambridge, UK; 2 School of Medicine, Anglia Ruskin University, Cambridge, UK; 3 Department of Computational Biomedicine, Cedars-Sinai Medical Center, Los Angeles, CA USA

**Keywords:** Breast cancer, Outcomes research

## Abstract

PREDICT Breast (www.breast .predict.nhs.uk) is a prognostication tool for early invasive breast cancer. The current version was based on cases diagnosed in 1999–2003 and did not incorporate the benefits of radiotherapy or the harms associated with therapy. Since then, there has been a substantial improvement in the outcomes for breast cancer cases. The aim of this study was to update PREDICT Breast to ensure that the underlying model is appropriate for contemporary patients. Data from the England National Cancer Registration and Advisory Service for invasive breast cancer cases diagnosed 2000–17 were used for model development and validation. Model development was based on 35,474 cases diagnosed and registered by the Eastern Cancer Registry. A Cox model was used to estimate the prognostic effects of the year of diagnosis, age at diagnosis, tumour size, tumour grade and number of positive nodes. Separate models were developed for ER-positive and ER-negative disease. Data on 32,408 cases from the West Midlands Cancer Registry and 100,551 cases from other cancer registries were used for validation. The new model was well-calibrated; predicted breast cancer deaths at 5-, 10- and 15-year were within 10 per cent of the observed validation data. Discrimination was also good: The AUC for 15-year breast cancer survival was 0.809 in the West Midlands data set and 0.846 in the data set for the other registries. The new PREDICT Breast model outperformed the current model and will be implemented in the online tool which should lead to more accurate absolute treatment benefit predictions for individual patients.

## Introduction

The PREDICT breast cancer prognostication and treatment benefit prediction model (v1) was developed in 2010 using data from the UK East Anglia Cancer Registration and Information Centre (ECRIC) for model fitting and data from the West Midlands Cancer Intelligence Unit for model validation^1–3^. The model fitting data set comprised data on 5232 cases diagnosed from 1999 to 2003. PREDICT v1 was implemented as a web-based tool for clinicians in January 2011 (www.breast.predict.nhs.uk), and since then the use of the tool has increased steadily around the world. The model was refitted in 2017 using the original cohort of cases from East Anglia with updated survival time in order to take into account age at diagnosis and to smooth out the hazard ratio functions for tumour size and node status (v2)^4^. PREDICT has been independently validated in cohorts from Canada^5^, Malaysia^6^, the Netherlands^7–9^, and the UK^10,11^ and has generally been shown to have good discrimination and calibration.

The data on which PREDICT breast v1 and v2 were based were breast cancer cases diagnosed in the Eastern Region of England over 20 years ago. Since then, the prognosis of early breast cancer has improved substantially^12^ and it is likely that the current model is not well calibrated for contemporary patients^13^. Moreover, the number of cases with ER-negative disease in the cohort was comparatively small (<1000) and it is possible that the estimates of the prognostic effects of the variables in the ER-negative disease model were sub-optimal. Furthermore, radiotherapy and chemotherapy have been shown to be associated with an increase in mortality from causes other than breast cancer^14,15^ and this was not taken into account in previous versions of PREDICT Breast.

We have therefore refitted the PREDICT breast model using a national data set of patients diagnosed from 2000 to 2017 with the aim of refining the hazard ratio estimates for the variables in the current model and to estimate the effect of the year of diagnosis on prognosis in order to be able to recalibrate the model for contemporary patients. In addition, we included the beneficial effect of radiotherapy on breast cancer mortality and the harmful effect of both chemotherapy and radiotherapy on other causes of mortality. Model development, validation and reporting were carried out according to the TRIPOD (Transparent Reporting of a multivariable prediction model for Individual Prognosis Or Diagnosis) criteria^16^.

## Results

Table 1 shows the patient characteristics by the cancer registry. The model fitting was carried out using Eastern Cancer Registry data for 4644 women with an ER-negative tumour and 34,265 women with an ER-positive tumour.

**Table 1:** Patient characteristics for the Eastern Cancer Registry, the West Midlands Cancer Registry and the other cancer registries (mean (sd), unless stated otherwise).

	Cancer Registry					
	Eastern		West Midlands		Other	
Age	59.9	(12)	60.1	(11.8)	60.4	(12)
Follow up time^a^, years	7.0	(4.0)	7.7	(4.3)	4.5	(2.2)
Tumour size, cm	2.1	(1.5)	2.0	(1.3)	2.1	(1.5)
*Tumour grade*, *n* (%)						
G1	5570	(16)	5986	(19)	16,900	18
G2	18,233	(51)	15,761	(50)	50,273	52
G3	11,671	(33)	10,202	(32)	28,776	30
*ER status*, *n* (%)						
Negative	4644	(13)	4668	(15)	12,814	13
Positive	30,830	(87)	27,133	(85)	83,135	87
*Node status*						
Negative	24,042	(68)	25,592	(81)	66,739	(70)
Positive	11,432	(32)	62.09	(20)	292,10	(30)
*Mode of detection*, *n* (%)						
Clinically detected	20,663	(58)	19,077	(60)	55,093	(57)
Screen detected	14,811	(42)	12,724	(40)	40,856	(43)
Chemotherapy, *n* (%)	11,939	(34)	10,823	(34)	33,259	(35)
Hormone therapy, *n* (%)	21,090	(60)	12,518	(40)	41,145	(43)
Radiotherapy, *n* (%)	23,801	(67)	22,114	(70)	56,704	(60)
*Vital status*, *n* (%)						
Alive	29,666	(84)	26,163	(82)	87,674	(91)
Died breast cancer	3099	(8.7)	2529	(8.0)	4512	(4.7)
Died other causes	2709	(7.6)	3109	(9.8)	3763	(3.9)

^a^Censored at 15 years follow up.

**Fig. 1:**
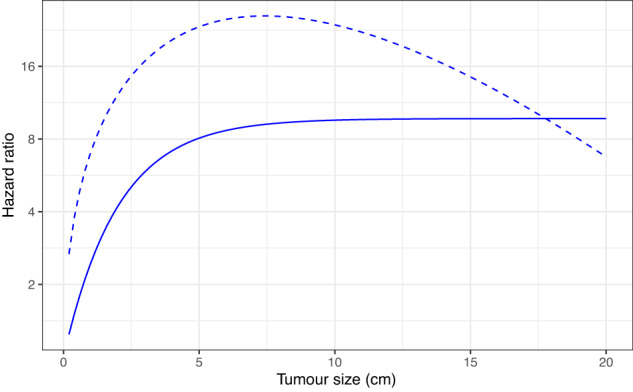
Polynomial hazard ratio functions for tumour size in ER-positive disease. Dashed line—best fit from the multivariable fractional polynomial model. Solid line—monotonic function selected for inclusion in the final model.

**Fig. 2:**
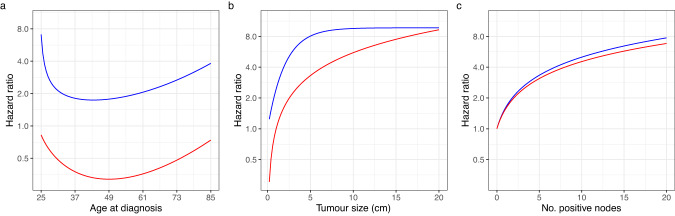
Breast cancer-specific mortality hazard ratio functions. **a** Age, **b** tumour size, and **c** the number of positive nodes. ER-negative is indicated by red lines and ER-positive is indicated by blue lines. Note that the hazard ratios for ER-negative and ER-positive disease should not be directly compared as an indicator of prognosis in ER-negative disease compared to ER-positive disease because the risk is a function of both the hazard ratio and the ER-status specific baseline hazards.

On fitting the multivariable fractional polynomial model to the ER-positive cases the hazard ratio function for tumour size was found to be 2.39*(size)^0.5^–0.439*size. Under this function, the hazard ratio would increase to a maximum for a tumour of 7.4 cm and then decrease for larger tumours (Fig. 1 dashed line). It seems unlikely that the true effect size would get smaller with increasing tumour size so we refitted the model using 1–exp(−size/2) so that the hazard ratio increases up to 7.5 cm and then flattens off (Fig. 1 solid line). The breast cancer-specific mortality hazard ratio (HR) functions for age at diagnosis, tumour size and number of positive nodes for the ER-negative and ER-positive cases are shown in Fig. 2 and the associated logarithmic hazard ratios in Table 2.

**Table 2:** Fractional polynomial functions and associated logarithmic hazard ratios for age at diagnosis, tumour size, number of positive nodes, tumour grade and mode of detection by oestrogen receptor (ER) status.

Prognostic factor	Function	Log HR	*p*-value
*ER-negative breast cancer-specific mortality*			
Age at diagnosis 1	((age-24)/100)	1.756	<0.0001
Age at diagnosis 2	((age-24)/100)*log((age-24)/100))	4.555	<0.0001
Tumour size, cm	log(size)	0.744	<0.0001
No. of positive lymph nodes	log(nodes+)	0.631	<0.0001
Tumour grade	Grade-1	0.346	<0.0001
Mode of detection	Screen detected	−0.211	0.037
Year of diagnosis	Year-2000	−0.046	<0.0001
*ER-positive breast cancer-specific mortality*			
Age at diagnosis 1	((age-24)/100)^−0.5^	0.196	0.0004
Age at diagnosis 2	((age-24)/100)^2^	2.929	<0.0001
Tumour size 1, cm	1–exp(−size/20)	2.274	<0.0001
No. of positive lymph nodes	log(nodes + 1)	0.672	<0.0001
Tumour grade	Grade-1	0.705	<0.0001
Mode of detection	Screen-detected	−0.320	<0.0001
Year of diagnosis	Year-2000	−0.048	<0.0001
*All cases non-breast cancer mortality*			
Age at diagnosis 1	((age-24)/100)^3^	4.21	0.0007
Age at diagnosis 2	((age-24)/100)^3^*log((age-24)/100))	−31.4	<0.0001
Year of diagnosis	Year-2000	−0.021	0.0001

The derived polynomial baseline hazard functions for breast cancer-specific mortality in the ER-negative cases, ER-positive cases, and non-breast cancer mortality are given by the following equations:

1 $$\begin{array}{l}{\rm{ER}}-{\rm{negative}}:{{\rm{baseline}}\; {\rm{hazard}}}=\exp \left(-3.015-0.576\times {\left(\frac{t}{10}\right)}^{-1}\right.\\\qquad\qquad\qquad\qquad\qquad\qquad\qquad\;\;\left.-0.103\times {\left(\frac{t}{10}\right)}^{-1}\times \log \left(\frac{t}{10}\right)\right)\end{array}$$

2 $$\begin{array}{l}{\rm{ER}}-{\rm{positive}}:{{\rm{baseline}}}\,{{\rm{hazard}}}=\exp \left(-2.319-3.623\times {\left(\frac{t}{10}\right)}^{-0.5}\right.\\\qquad\qquad\qquad\qquad\qquad\qquad\qquad\left.-0.542\times {\left(\frac{t}{10}\right)}^{-0.5}\times \log \left(\frac{t}{10}\right)\right)\end{array}$$

3 $$\begin{array}{l}{\rm{Non}}-{\rm{breast}}\; {\rm{mortality}}:{{\rm{baseline}}}\,{{\rm{hazard}}}={\rm{exp}} \left(-4.846+1.341* \log \left(\frac{t}{10}\right)\right.\\\qquad\qquad\qquad\qquad\qquad\qquad\qquad\qquad\qquad\left.+0.495* \left(\frac{t}{10}\right)\right)\end{array}$$

These functions provided a very good fit to the observed baseline hazard (Supplementary Fig. 1).

### Model calibration

Table 3 shows the cumulative number of breast cancer deaths predicted at 5, 10, and 15 years by the new version of the model (v3.0) and the current version of the model (v2.2) by cancer registry and ER status. As expected, for breast cancer-specific mortality, v3.0 is well-calibrated in the model development data. It also performs well in the two validation data sets; in all strata of the data, the predicted number of deaths was within 10% of that observed. In contrast, v2.2 consistently over-predicted the number of deaths as might have been expected given the general improvement in prognosis observed since the data on which v2.2 was generated. Prediction of non-breast cancer mortality by v3.0 (Table 4) was also excellent in the model development data, but under-predicted by about 10% in the validation data sets. Again, v2.2 substantially overpredicted other mortality in all the data sets.

**Table 3:** Cumulative observed versus predicted breast cancer deaths estimated by the updated version of PREDICT Breast (v3.0) and the current version (v2.2) by cancer registry and ER status at up to 5, 10, and 15 years follow up.

Cancer registry		No. of cases	Observed	Predicted		Predicted–expected (%)			
				v3.0	v2.2	v3.0		v2.2	
*5-year mortality*									
Eastern	ER-	5484	908	883	1150	−25	(−3)	242	(27)
	ER+	34,265	1354	1247	1659	−107	(−8)	305	(23)
West Midlands	ER-	4734	672	642	871	−30	(−5)	199	(30)
	ER+	27,674	900	858	1164	−42	(−5)	264	(29)
Others	ER-	13,369	1643	1560	2377	−83	(−5)	734	(45)
	ER+	87,182	2228	2262	3527	34	(2)	1299	(58)
*10-year mortality*									
Eastern	ER-	5484	1123	1091	1331	−32	(−3)	208	(19)
	ER+	34,265	2385	2335	2939	−51	(−2)	554	(23)
West Midlands	ER-	4734	810	807	1022	−3	(0)	212	(26)
	ER+	27,674	1509	1600	2040	91	(6)	531	(35)
Others	ER-	13,369	1789	1715	2533	−74	(−4)	744	(42)
	ER+	87,182	2865	2963	4472	98	(3)	1607	(56)
*15-year mortality*									
Eastern	ER-	5484	1155	1120	1349	−35	(−3)	194	(17)
	ER+	34,265	2732	2705	3339	−27	(1)	607	(22)
West Midlands	ER-	4734	826	835	1041	9	(1)	215	(26)
	ER+	27,674	1725	1882	2346	157	(9)	621	(36)
Others	ER-	13,369	1793	1717	2535	−76	(−4)	742	(41)
	ER+	87,182	2890	2983	4494	93	(3)	1604	(55)

**Table 4:** Cumulative observed versus predicted deaths from other causes estimated by the updated version of PREDICT Breast (v3.0) and the current version (v2.2) by cancer registry at up to 5, 10 and 15 years follow up.

	No cases	Observed	Predicted		Predicted – expected (%)			
			v3.0	v2.2	v3.0		v2.2	
*5-year mortality*								
Eastern	39,749	1201	1195	1784	26	(−0.5)	583	(49)
West Midlands	38,999	1135	983	1466	−152	(−13)	331	(29)
Others	32,408	2886	2495	4055	−391	(−14)	1169	(41)
*10-year mortality*								
Eastern	39,749	2450	2495	3282	45	(2)	832	(34)
West Midlands	38,999	2350	2128	2791	−22	(−9)	441	(19)
Others	32,408	3832	3354	5153	−478	(−13)	1321	(34)
*15-year mortality*								
Eastern	39,749	3111	3219	3977	108	(43)	866	(28)
West Midlands	38,999	3125	2801	3446	−324	(−10)	321	(10)
Others	32,408	3861	3385	5184	−476	(−12)	1323	(34)

**Fig. 3:**
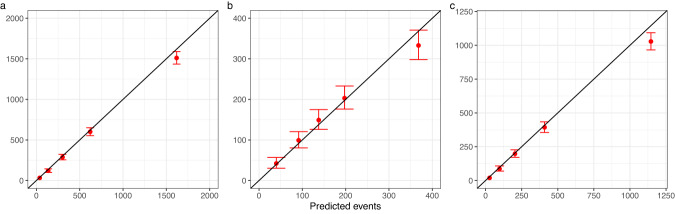
Observed and predicted breast cancer deaths at 15 years in West Midlands data set by quintile of predicted risk. **a** All cases, b ER-negative cases, and **c** ER-positive cases.

The observed and predicted breast cancer deaths in the West Midlands cancer registry by quintile of predicted risk for the updated version of PREDICT Breast are shown in Fig. 3 which shows that calibration is excellent at all levels of risk.

### Model discrimination

Model discrimination (area under the receiver operator characteristic curve) was good in all strata of the data (Table 5). In general, the model for ER-positive disease performed better than that for ER-negative disease and the performance of the model in the model development data from the Eastern Cancer Registry was slightly better than the performance in the two validation data sets. PREDICT v3.0 performed consistently slightly better than v2.2.

**Table 5:** The discrimination for up to 5-year, 10-year and 15-year breast cancer-specific mortality by cancer registry and ER status.

Cancer Registry	ER status	5-year		10-year		15-year	
		v3.0	v2.2	v3.0	v2.2	v3.0	v2.2
Eastern Region	ER+	0.843	0.837	0.821	0.809	0.836	0.833
	ER−	0.771	0.764	0.774	0.766	0.778	0.773
	All	0.824	0.819	0.813	0.802	0.833	0.832
West Midlands	ER+	0.831	0.826	0.804	0.793	0.812	0.811
	ER−	0.735	0.726	0.719	0.710	0.717	0.716
	All	0.809	0.806	0.795	0.782	0.811	0.809
Other	ER+	0.861	0.857	0.856	0.849	0.865	0.862
	ER−	0.777	0.771	0.777	0.770	0.783	0.777
	All	0.846	0.844	0.847	0.842	0.858	0.857

### Model reclassification

The Cambridge Breast Unit classifies women with breast cancer into three groups based on the predicted benefit of adjuvant chemotherapy at 10 years as given by the absolute reduction in risk of breast cancer-specific mortality; low-risk women are those with a predicted 10-year benefit of 0–3% who would usually be advised not to have adjuvant chemotherapy and high-risk women are those with a predicted benefit of over 5% who would usually be advised to have adjuvant chemotherapy^17^. The advice to intermediate-risk women (3–5%) would depend more on other factors including patient preferences. While the benefit of therapy depends on patient age and adjuvant chemotherapy regime it is possible to classify women into similar categories based on the predicted breast cancer mortality at 10 years: low risk being 0–15%, medium risk being 15–20% and high-risk being >20% risk of breast cancer death at 10 years. Based on these risk categories it is possible to evaluate reclassification comparing PREDICT v3.0 with v2.2. Of 32,408 breast cancer cases in the West Midlands data set 4203 (13%) women would be classified in different risk groups by PREDICT v2.2 and v3.0 (Table 6).

**Table 6:** Re-classification of 32,408 West Midlands Cancer Registry breast cancer cases by PREDICT v3.0 into low-, medium- and high-risk compared to PREDICT v2.2 classification.

PREDICT v2.2		PREDICT v3.0		
	Low risk	Medium risk	High risk	Total
Low risk	22,132	216	7	22,355
Medium risk	2902	791	157	3852
High risk	919	1533	3749	6201
Total	25,953	2540	3915	32,408

## Discussion

We have used data from the National Cancer Registration and Analysis Service for England for breast cancer cases diagnosed from 2000 to 2017 to develop and validate a new PREDICT Breast prognostic model (v3.0). We used a similar analytic approach to that used to develop PREDICT Breast v2.0 using multi-variable fractional polynomials within a Cox regression framework to create different models for breast cancer-specific mortality for ER-positive disease and ER-negative disease and non-breast cancer mortality. The major difference between v2.2 and v3.0 is that v3.0 includes a term for year of diagnosis as the data show a clear trend from improved survival rates over time.

It has previously been observed that the log hazard ratio function for age at diagnosis in ER-positive breast cancer is U-shaped with breast cancer in young women and older women being associated with a poorer prognosis. However, a similar relationship in ER-negative disease has not been previously described—age at diagnosis in v2.2 was modelled as a linear term. However, in this much larger data set, we also observed a U-shaped function for age at diagnosis in ER-negative disease. We also observed an unexpected hazard ratio function for tumour size in ER-positive cases with an inverted U-shape. There may be a biological reason for this—it is conceivable that for tumours to become very large in size they would need to be growing for a long time without metastasizing, and so may be inherently less aggressive. However, despite our very large data set, the number of ER-positive cases with tumours above 7.5 cm was only 414 with 80 deaths from breast cancer and the precision of the hazard ratio estimates in larger tumours will be small. We therefore chose to constrain the polynomial function such that the hazard ratio flattened off but did not get smaller with increasing tumour size.

Overall, the model performed well in terms of discrimination and calibration in both model development data and the model validation data. We assumed that all patients receiving chemotherapy received a standard-dose anthracycline-based regime and all patients receiving hormonal therapy received 5-year treatment. However, some patients will have received taxane-based or high-dose anthracycline-based chemotherapy, and some patients will have had 10 years of hormonal therapy. Similarly, we assumed the same benefit for all patients treated with radiotherapy whereas whole breast irradiation alone after breast-conserving surgery is likely to have different effects than post-mastectomy radiotherapy to the chest wall and regional nodes. Furthermore, mortality from causes other than breast cancer was modelled as a function of age and therapy and we assumed similar relative harm for chemotherapy and radiotherapy, although this may vary by the type of chemotherapy and radiotherapy received. Non-breast cancer mortality is also affected by co-morbidities and lifestyle factors such as smoking. Other factors could not be included in the models because information on co-morbidities is not available in the NCRAS data. These misclassifications would not be expected to affect model calibration but would be likely to reduce discrimination.

The improvement in prognosis over time is reflected in the reclassification of breast cancer cases within the three categories of risk used by the Cambridge Breast Unit to guide the use of adjuvant chemotherapy. In the West Midlands data set 10,053 cases would be classified as moderate or high risk by PREDICT Breast v2.2 and would be considered candidates for adjuvant chemotherapy. Of these, 3,821 (38%) would be reclassified as low risk by PREDICT Breast v3.0 and spared the harms of chemotherapy. The reason for the improvement in prognosis over time is not clear—the effect of the year of diagnosis is seen after adjusting for the known major prognostic factors and after adjusting for treatment. General improvements in the organization and standardization of cancer services with better targeting of systemic therapies and improvements in the delivery of radiotherapy are likely to play a role. Some improvement will be due to the increased use of therapies such as trastuzumab and bisphosphonates and improved management of disease relapse with second-line therapies.

Tumour gene expression profile tests (also known as genomic risk scores) are being increasingly used to guide treatment decisions in breast cancer^18^. The results of genomic risk scores are not available in the cancer registration data set used for these analyses and it was not possible to assess any added value of such scores to PREDICT v3.0. However, it has been shown that genomic risk scores do not significantly improve the discrimination of PREDICT v2.2^19^. Further research to evaluate the performance of genomic risk scores by themselves and in combination with other biomarkers such as KI67 in breast cancer patients shown to be at intermediate risk by PREDICT v3.0 is warranted.

Another limitation of this model is that it does not include either local or distant recurrence as an endpoint, as these data are not available in the NCRAS data set. While mortality as an endpoint is important in decision-making, recurrence may also be an important end point for some patients. In the future, the integration of electronic health records with cancer registration data may enable the accurate encoding of recurrence and the inclusion of other endpoints in the model.

In an era of precision oncology, accurate, well-validated models that predict patient outcomes are invaluable clinical tools. We have derived an improved version of the PREDICT prognostication and treatment benefit model to reduce some of the limitations of the current version. In particular, we have included updated the model to reflect outcomes in contemporary patients and added the benefits of radiotherapy as well as the harms of both chemotherapy and radiotherapy. The new model has been validated in two independent population-based data sets from the United Kingdom and performs well. It will be implemented in the online tool available at www.breast.predict.nhs.uk and will continue to aid clinical decision-making in clinical practice.

## Methods

### Patient data

The study was approved by the Public Health England Office for Data Release. Public Health England provided anonymized data from the National Cancer Registration and Analysis Service (PHE NCRAS) for all women diagnosed in England with non-metastatic invasive breast cancer from 2000 to 2017 inclusive. Ethical approval by the National Research Ethics Service was not required because all analyses were carried out on an anonymized data set provided by Public Health England. Information obtained from PHE NCRAS included age at diagnosis, year of diagnosis, tumour size, histological grade, tumour stage at diagnosis, number of lymph nodes sampled, number of lymph nodes positive, ER status, HER2 status, mode of detection (clinically detected vs. screen-detected), and whether the patient had undergone chemotherapy, hormone therapy and/or radiotherapy for two time periods, the first being within 6 months following their diagnosis. Data on other biomarkers such as tumour KI67 expression status were not available in the NCRAS data set. Patients younger than 25 or older than 85 at diagnosis, patients with a tumour larger than 20 cm, or with more than 20 positive lymph nodes were excluded from the analysis. Of 372,110 cases, complete data were available for 163,224 (44%). Initial analyses showed that the Eastern Cancer Registry and the West Midlands Cancer Registry had fewer missing data (62% and 71% complete cases) compared to the other registries (35% complete cases), particularly in the years 2000–2009 (Supplementary Table 1). The variable with the most missing data was ER status (42% missing), 31% were missing the number of positive nodes, 16% were missing tumour size, 3% were missing tumour grade and 6% were missing mode of detection. The complete case data set for the Eastern Cancer Registry (*n* = 35,474; 4644 ER-negative and 30,830 ER-positive ) was used for the development of the new version of PREDICT Breast and the West Midlands Cancer Registry data set (*n* = 31,801; 4668 ER-negative; 27,133 ER-positive) was used as the primary validation data and the data set for the other cancer registries (*n* = 95,949; 12,814 ER-negative; 83,135 ER-positive) used as an additional validation data set.

Details of the specific regimen used for radiotherapy, chemotherapy, duration of hormonal therapy, or use of trastuzumab or bisphosphonates were not available. We assumed that all patients who underwent chemotherapy were treated with an anthracycline-based regimen and that all women received hormonal therapy for 5 years. The benefits of radiotherapy were applied to all patients who received including those who had lumpectomy and those who had mastectomy as the primary surgical treatment. Death certificate flagging through the Office for National Statistics provides the registries with notification of deaths. The lag times for these are a few weeks for cancer deaths and 2 months to 1 year for non-cancer deaths. Vital status was ascertained at the end of December 2019, and so all analyses were censored on 31 December 2018 to allow for a delay in reporting vital status. Breast cancer-specific mortality was defined as deaths where breast cancer was listed as the cause of death on parts 1a, 1b or 1c of the death certificate.

### Statistical methods

Multivariable Cox proportional hazards models were used to estimate the prognostic effect of each variable. In all models, follow-up time was defined as the time from breast cancer diagnosis to the last follow-up, death or 15 years after diagnosis, whichever came first. The outcome of interest was either breast cancer-specific mortality or mortality from other causes.

Separate models were derived for breast cancer-specific mortality in ER-negative and ER-positive cases. Multiple fractional polynomials were used to model non-linear effects between the continuous risk factors (age at diagnosis, tumour size and number of positive nodes) and breast cancer-specific mortality as adding higher order polynomials to the model will improve the fit to the data in the presence of non-linearity. Sequential backward elimination with a maximum of 4 degrees of freedom for a single continuous predictor was used to estimate the continuous variable transformations. In addition to the variables already present in the current version of PREDICT, the year of breast cancer diagnosis and the effect of radiotherapy were also incorporated into the analyses. Age at diagnosis was transformed to age at diagnosis minus 24 and year of diagnosis was transformed to year minus 2000 in order that the baseline hazard would be more realistic. The baseline hazard is the hazard that corresponds to a hypothetical individual with all variables taking a value of zero. Transforming age at diagnosis and year at diagnosis in this way means that the baseline hazard corresponds to a woman diagnosed at age 24 in the year 2000 rather than a woman diagnosed at age 0 in the year 0. The relative treatment benefits for chemotherapy, hormone therapy, and radiotherapy were constrained to the estimates of benefit randomized controlled trial meta-analyses of the Early Breast Cancer Trialists Collaborative Group (adjuvant hormone therapy log hazard ratio −0.386^20^, adjuvant chemotherapy log hazard ratio −0.248^21^, radiotherapy log hazard ratio −0.180^22^) by adding them as an offset in the analyses. After fitting the Cox proportional hazards models to ER-negative and ER-positive cases, a multiple fractional polynomial model with a Gaussian distribution was fit to the baseline hazards according to the method of Sauberei and colleagues^23^ to derive a smoothed baseline hazard functions for breast cancer-specific mortality.

A single multivariate Cox regression model for mortality from other causes (non-breast cancer-specific) was built for ER-negative and ER-positive cases combined with year of diagnosis and age at diagnosis modelled using multivariable fractional polynomials. The relative harms of chemotherapy and radiotherapy were constrained to the estimates reported by Kerr and colleagues (adjuvant chemotherapy log hazard ratio 0.183)^14^ and Taylor and colleagues (radiotherapy log hazard ratio 0.078 per Gray whole-heart dose)^15^ by adding them as an offset in the analyses. We assumed all patients receiving radiotherapy receive a whole heart dose of 2 Gy, as the radiotherapy dose was not available in our data. The smoothed baseline hazard function for non-breast cancer-specific mortality was also computed using a multivariable fractional polynomial model.

### Model validation

The models derived from the Eastern Cancer Registry were used to predict the probabilities of death from breast cancer or death from other causes in the cases in both validation data sets. Because the web version of PREDICT Breast v2.2 allows for missing data on the mode of detection we also included 9848 cases for whom only modes of detection were missing. Model calibration was performed by comparing the observed number of deaths with those predicted by v3.0 and v2.2 up to 5 years, 10 years and 15 years after diagnosis. Calibration plots were used to visualize calibration at different levels of risk. Model discrimination was evaluated by calculating the area under the receiver operator-characteristic curve (AUC) for up to 5-year, 10-year and 15-year breast cancer mortality. The AUC is the probability that the predicted mortality from a randomly selected patient who died will be higher than the predicted mortality from a randomly selected survivor.

The study has been reported in accordance with the TRIPOD guidelines for reporting a multivariable prediction model for individual prognosis^16^. All analyses were carried out using the *mfp*^24^, *patchwork*^25^, *pROC*^26^, *survival*^27^, *tableone*^28^ and *tidyverse*^29^ packages for the R software^30^ implemented in R Studio^31^.

### Reporting summary

Further information on research design is available in the Nature Research Reporting Summary linked to this article.

### Supplementary information

Supplemental material

Reporting summary

## Data Availability

The data used for these analyses cannot be shared by the authors for reasons of confidentiality. They are available on request from the England National Disease Registration Service at https://digital.nhs.uk/services/national-disease-registration-service#requests-for-access-to-ndrs-data.

## Code availability

All analyses were carried out using the *mfp*^24^, *patchwork*^25^, *pROC*^26^, *survival*^27^, *tableone*^28^, and *tidyverse*^29^ packages for the R software^30^ implemented in R Studio^31^. The R scripts used to analyse the data and draw the figures are available from the corresponding author on request.
