# Supplementary material for: An updated PREDICT breast cancer prognostic model including the benefits and harms of radiotherapy
Source: NPJ Breast Cancer. 2024 Jan 15;10:6. doi: 10.1038/s41523-024-00612-y (PMC10789872; doi:10.1038/s41523-024-00612-y)
Supplement: Supplementary file 1 — Supplemental material [file 41523_2024_612_MOESM1_ESM.pdf]

SUPPLEMENTARY MATERIAL

Supplementary Table 1 Number of breast cancer cases by year of diagnosis and cancer registry with associated data missingness

Year	No of cases	Complete cases (%)	No cases missing data by variable					
			Any	ER status	Grade	Size	Nodes	Mode of detection
Eastern Cancer Registry								
2000	1664	47	888	370	143	87	231	461
2001	1747	49	894	332	123	121	230	514
2002	1946	53	919	303	163	118	262	532
2003	2071	50	1027	456	156	153	336	510
2004	2196	37	1377	935	140	165	344	497
2005	2872	34	1897	1179	176	208	474	726
2006	3379	41	1993	1107	184	216	529	744
2007	3311	58	1375	336	161	143	468	733
2008	3562	40	2122	1690	163	112	435	297
2009	3563	77	803	259	123	156	444	118
2010	3612	82	648	149	96	102	445	74
2011	3499	79	728	101	82	115	556	63
2012	3744	74	972	145	103	102	637	203
2013	3909	74	1004	159	80	159	728	153
2014	3967	71	1156	247	85	245	752	167
2015	4073	72	1125	174	71	233	790	169
2016	4016	67	1340	277	85	200	894	274
2017	3896	67	1285	278	47	164	986	83
West Midlands Cancer Registry								
2000	1618	8	1494	1486	64	61	43	1
2001	1688	11	1502	1486	52	49	38	3
2002	1692	87	228	106	53	48	51	2
2003	1838	88	222	120	51	54	23	2
2004	1930	92	148	75	36	35	23	1
2005	2122	90	209	93	50	65	42	0
2006	1956	91	172	77	45	49	26	1
2007	2073	93	140	49	51	40	22	0
2008	2142	92	168	39	49	74	37	1
2009	2054	91	184	37	46	91	32	1
2010	2246	87	283	90	42	125	63	2
2011	3175	81	611	195	86	228	248	9
2012	3042	74	782	352	66	277	395	14
2013	3272	63	1209	534	85	361	594	106
2014	3508	63	1304	506	90	451	669	136
2015	3459	57	1476	493	91	613	664	229
2016	3399	60	1343	388	55	410	710	356
2017	3480	59	1418	561	60	505	809	76

Year	No of cases	Complete cases (%)	No cases missing data by variable					
			Any	ER status	Grade	Size	Nodes	Mode of detection
Other Cancer Registries								
2000	8496	0	8469	8440	651	2542	6535	1171
2001	10112	0	10068	10047	693	2928	6972	1671
2002	10840	1	10785	10729	638	2646	6695	1873
2003	12712	1	12643	12604	681	2974	7675	1841
2004	8509	1	8436	8388	410	2582	6732	726
2005	8945	1	8892	8856	367	2814	7159	246
2006	8986	1	8929	8901	425	3173	6770	137
2007	9010	1	8951	8914	335	2880	7113	294
2008	8748	1	8679	8645	199	1742	4045	178
2009	9545	4	9170	9079	234	664	2552	73
2010	10359	35	6776	6186	245	1101	2521	95
2011	15079	66	5109	3308	360	1607	2842	185
2012	22439	61	8803	5290	708	3286	4544	351
2013	23117	62	8764	4233	437	3305	4302	1020
2014	25231	56	11010	5551	550	4463	5729	1202
2015	25926	53	12107	5858	397	5534	5511	1509
2016	26171	47	13753	7718	337	4271	5563	3132
2017	26164	50	13096	7956	360	4237	6485	866

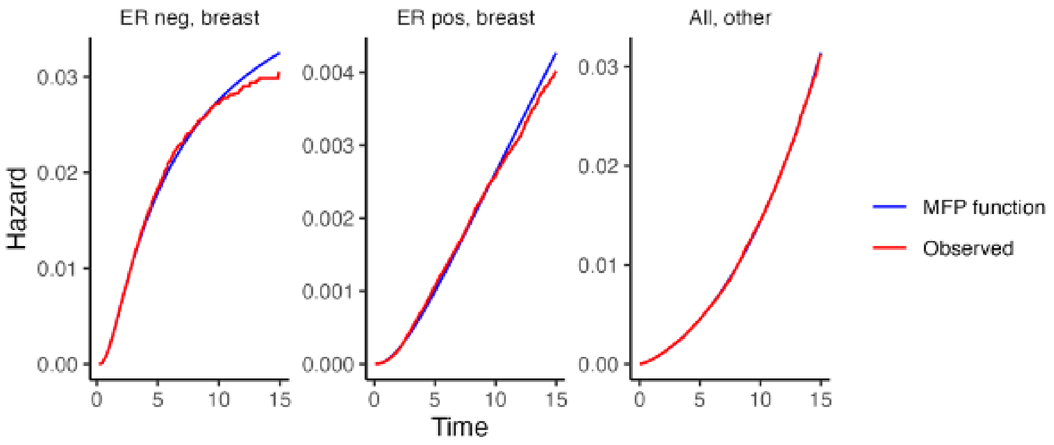

Supplementary Figure 1: Observed baseline hazard and fitted polynomial baseline hazard function for ER-positive breast cancer specific mortality, ER-negative breast cancer specific mortality and non-breast cancer mortality
